# One-Pot, In-Situ Synthesis of 8-Armed Poly(Ethylene Glycol)-Coated Ag Nanoclusters as a Fluorescent Sensor for Selective Detection of Cu^2+^

**DOI:** 10.3390/bios10100131

**Published:** 2020-09-23

**Authors:** Xiaoyuan Zhang, Guanghua Zhang, Gang Wei, Zhiqiang Su

**Affiliations:** 1 Beijing Key Laboratory of Advanced Functional Polymer Composites, State Key Laboratory of Chemical Resource Engineering, Beijing University of Chemical Technology, Beijing 100029, China; 2020700036@mail.buct.edu.cn (X.Z.); 2018210201@mail.buct.edu.cn (G.Z.); 2 College of Chemistry and Chemical Engineering, Qingdao University, Qingdao 266071, China

**Keywords:** hydrogel, silver nanoclusters, fluorescent sensor, Cu^2+^ ion, bionanomaterials, environmental science

## Abstract

Fluorescent nanomaterials, such as quantum dots, have developed rapidly in recent years and have been significantly developed. Herein, we demonstrate a facile, one-pot, and in-situ synthesis strategy to obtain fluorescent silver nanoclusters (AgNCs) coated with eight-armed poly (ethylene glycol) polymers (8PEG-AgNCs) via a direct gel-mediated process. During the synthesis, ammonium (NH_3_) served as the crosslinker for the gel formation via a amine-type Michael addition reaction. This hydrogel can be used as a template to synthesize AgNCs using its volume-limiting effect. The in-situ generation of AgNCs takes place inside the nanocages of the formed gels, which guarantees the homogenous distribution of AgNCs in the gel matrix, as well as the efficient coating of PEG on the nanoclusters. After the degradation of gels, the released 8PEG-AgNCs nanohybrids showed strong blue fluorescence and exhibited long-term stability in aqueous solution for nearly one year. Results showed that the fabricated sensor revealed excellent fluorescent sensitivity for the selective detection of Cu^2+^ with a detection limit of 50 nM and a wide linear detection range of 5–100 μM. It is proposed that the greater cross-linking density leads to smaller gel pores and allows the synthesis of AgNCs with fluorescent properties. These results indicate that this novel hydrogel with certain biodegradation has the potential to be applied as a fluorescent sensor for catalytic synthesis, fluorescence tracing in cells, and fluorescence detection fields. Meanwhile, the novel design principle has a certain versatility to accelerate the development and application of other kinds of metal nanoclusters and quantum dots.

## 1. Introduction

In the past few decades, fluorescent metal nanoclusters (usually less than 2 nm in diameter), consisting of several to a few hundred atoms, have been widely utilized in the fields of biosensing, bioimaging, as well as diagnostics [1,2,3,4,5,6], due to their facile synthesis, strong photoluminescence and high photostability [7,8]. In particular, silver nanoclusters (AgNCs) exhibit stronger fluorescence than other metal nanoclusters, and possess some unique applications, such as in antimicrobials [9,10,11]. However, AgNCs in aqueous solution are highly susceptible to oxidization, and easily undergo aggregation to form plasmonic particles at ambient temperatures [12,13,14]. Therefore, various additives, such as thiolates [15], dendrimers [16], polymers [17], and some biomolecules, including peptides [18], proteins [19], and DNA [20], have been employed to synthesize and stabilize AgNCs. The use of polyethylene glycol (PEG) long chains [21] or branched chains such as star polymers [22] as templates to prepare nanomaterials as a common method has often been reported in the previous literature. For PEG hydrogels used as templates, the stability and biodegradability of the hydrogels for gel formation are particularly important. In the same way, commonly used template materials, such as glass [23], DNA [24,25], polymethacrylic acid [26], peptides [18], and micelles [27], were utilized for preparing quantum dots. In these cases, the hydrogel materials can achieve size control of quantum dots through a certain volume-limiting effect [27]. 

Although many effective works have been carried out, there are still some challenges in this research field. For instance, additional reducing agents were required in some cases, such as Hydrazine hydrate [28]. This kind of reducing agents are very dangerous to human health [29]. Furthermore, the long-term stability was not acceptable and the control during nanocluster growth was absent. Moreover, the biocompatibility against fibroblast cells and the biodegradation ability of the materials were poor [30,31,32]. 

Recently, hydrogels, which are insoluble in water yet capable of absorbing water and exhibit three-dimensional (3D) network structures and are composes of molecular nanocages, have been used to control the nucleation and growth of the nanoclusters [22,33,34,35,36,37]. The hydrogel material as a template to prepare nanomaterials such as quantum dots should also have the following properties: (i) The template is able to maintain its properties stable during the reaction process. (ii) It is able to provide the physical and chemical effects necessary for crystal growth such as a limiting effect to limit the crystal volume growth, a certain coordination complexation effect to stabilize the nucleus, a suitable reaction environment, a uniform and slow reduction to prevent agglomeration. (iii) The prepared quantum dot material can be easily separated out. So far, the only hydrogel templates that can fully meet the above conditions are the peptide hydrogel templates formed by self-assembly [38] and the phenolic templates with higher strength [39], etc. However, these materials require strictly controlled reaction conditions, and cannot be used as a general and extensive template to prepare quantum dot materials. 

Cage dimensions and kinetic parameters of 3D hydrogels are key factors to mediate the nanocluster growth and yield the desired size of nanoclusters. For example, Chakraborty et al. reported reducing Ag(I) thiolates to form AgNCs in polyacrylamide gels’ nanocages [36]. In their method, the monomers containing Ag(I) thiolates polymerized to form a gel; afterwards, the reducing agent, aqueous NaBH_4_, was added, and then the color changed from light yellow to dark brown, indicating the formation of AgNCs inside of the nanocages. Although the monodisperse AgNCs were obtained, most of the formed clusters were trapped inside the gel and the released clusters lacked polymer modification due to the weak degradation ability of the gel matrix. 

**Figure 1:**
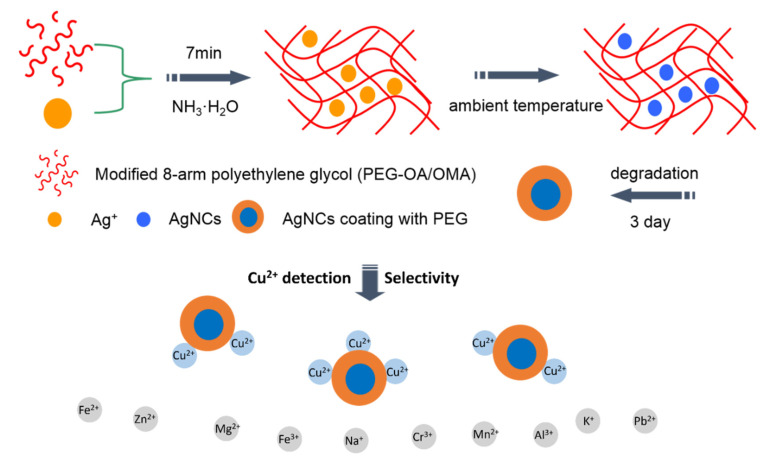
Schematic description for synthesizing fluorescent silver nanoclusters (AgNCs) coated with 8-armed poly (ethylene glycol) (8PEG) polymers and selective detection for Cu^2+^ ions.

So far, there is no work regarding hydrogel materials prepared from PEG as nanoscale reactors and templates for AgNCs. Thus, we proposed and developed a novel, one-pot and low-cost method for in-situ synthesizing of AgNCs with long-term stability, uniform size, and strong photoluminescence via a gel-mediated process (Figure 1). As the gelation is proceeding, the growth of AgNCs takes place at the same time without additional reducing agent. Unlike other gel template, our gels could be totally degraded in a few days and release eight-armed poly(ethylene glycol) (8PEG)-coated AgNCs, which are still finely dispersed in aqueous solution and exhibit bright fluorescence after one year storage at room temperature in a glass bottle. Moreover, these luminescent 8PEG-AgNCs nanohybrids are selectively sensitive to Cu^2+^ through fluorescence quenching, which can serve as an effective fluorescent sensor for Cu^2+^ ion detection. The novel gel-mediated method to a prepare polymer–NCs hybrid system with a versatile architecture, long-term stability and unique NC releasing ability via degradation opens a new avenue for designing advanced polymer–NCs functional materials.

## 2. Materials and Methods

### 2.1. Materials

Eight-armed poly(ethylene glycol) (8PEG-OH, Mw 15 KDa) was purchased from Jenkem technology USA. Eight-arm poly(ethylene glycol) acrylate (8PEG) was prepared via the same procedure as we have reported before [1]. Polyethylene glycol diacrylate (PEGDA, Mw = 2 kDa), 2-isocyanatoethylmethacrylate (purity 98%) and acryloyl chloride (purity 97%) were from TCI (Tokyo, Japan), tetrahydrofuran (THF, super dry solvent), ammonia water (37% *w/w*), alkaline aluminum oxide, dibutyltin dilaurate were purchased from J&K (Beijing, China). All the metal ions were in the nitrate form and were purchased from Beijing Chemical Co. (Beijing, China). Other reagents were purchased from Aldrich and used as received unless stated otherwise. Solvents were at least analytical grade quality.

### 2.2. Synthesis of Acryloyl Chloride Modified 8-Armed Polyethylene Glycol (PEGOA)

First, 8PEG-OH (5 g) with a hydroxyl capped end was synthesized at 60 °C in a dried vacuum oven for 4 h. 8PEG-OH was dissolved in 50 mL of dichloromethane in a super dry solvent under nitrogen atmosphere, and then placed into the solution in an ice water bath. Then 4 g of sodium carbonate was added to the mixture to neutralize the esterification reaction to produce the acid. The solution was stirred and reacted with a magnetic stirrer at 35 °C under a continuous nitrogen flow for 4 days. The reacted solution was filtered through an alkaline alumina column to remove the insoluble solids and the acid generated during the reaction. The solution was evaporated by vacuum at 20 °C, until a certain concentration and then dropped into an excess of cooled anhydrous ethyl ether (<4 °C). The sediment was collected in a centrifuge tube with constant and rapid stirring and centrifuged (5000 rpm for 15 min). The lower layer was precipitated, and the precipitate was dried in a vacuum drying oven at 40 °C for 6 h to obtain a white powdery solid, and the yield was calculated after weighing. The reduction rate was measured to be 72%. 

### 2.3. Synthesis of 8-Armed Polyethylene Glycol (PEGOMA) Capped with Methacrylate (2-Isocyanoethyl) Ester

The 8PEG-OH (2 g) was dissolved in 50 mL of THF. An amount of 0.6 mL of methacrylic acid (2-isocyanoethyl) ester was added to THF super-dry solvent by pipetting with vigorous agitation. An amount of 30 μL of dibutyltin dilaurate was used as the catalyst. The solution was bubbled under nitrogen for 10 min and then reacted in a water bath at 60 °C for 20 h. The solution was evaporated in a vacuum at 30 °C until about 10 mL of solvent remained. The solution was evaporated in a vacuum at 30 °C until about 10 Ml of solvent remained, and then the solution was added drop by drop to a 150 μM solution. The ether was used to obtain a suspension containing a white floc-like precipitate. The precipitate and the suspension were centrifuged at high speed for 15 min (5000 rpm). The supernatant was discarded, and the bottom was vacuum-dried at 30 °C. After 2 h of drying in a drying chamber, the desired product was obtained as a white bulk solid with a yield of 94% after weighing. 

### 2.4. Determination of the Critical Gelation Concentration of PEGOMA and PEGOA Hydrogels

The critical gelation concentrations of PEGOMA and PEGOA hydrogels were determined by the inversion method. The purified PEGOMA and PEGOA were weighed accurately, and 0.1–3 mL of each was added. A volume of primary water, sonicated for half an hour in an ultrasonic cleaner, was used to dissolve the sample from different concentrations and dissolved in a 5 mL centrifuge tube. An amount of 50 μL of ammonia was added separately with a pipette gun and then quickly the solution was ultrasonicated for 1 h. The critical gelling concentration is the minimum concentration at which the solution stops flowing after tilting or inverting the test tube.

### 2.5. Determination of Gelation Time of PEGOMA and PEGOA Hydrogels

The gelation time of the hydrogels was determined by the inversion method, and 150 mg of PEGOMA and PEGOA were weighed multiple times. The hydrogels were dissolved in 150 mL of primary water and sonicated for half an hour to obtain a clear solution. An amount of 30 μL of ammonia was added at different concentrations to the solution by pipetting, then the tube was shaken rapidly and placed in the ultrasonic washer for 10 s. Simultaneously, a stopwatch was started. The tube was tilted or inverted every few seconds to obtain the PEGOMA and PEGOA solutions. 

### 2.6. In-Situ Synthesis of AgNCs Using 8PEG-NH_3_ Hydrogel as Template

After mixing 150 mg of 8PEG polymer with certain amounts of distilled water in a 5 mL plastic centrifuge tube, 20 μL of AgNO_3_ solution (100 mg/mL in water) was injected and then ultrasonicated for 20 min to form a homogenized solution. Then, 50 μL of ammonia solution was injected. The tube was violently shaken and then ultrasonicated for 2 min to remove bubbles. The solution turned into hydrogel in 5–30 min according to the concentration of 8PEG solution. Colorless and fluorescent 8PEG-AgNCs solution could be obtained after the degradation process. 

### 2.7. Detection of the Cu^2+^ Ions Using the 8PEG-AgNCs Nanohybrids

A typical Cu^2+^ detection procedure was performed as follows. Copper nitrate was utilized for the study of Cu^2+^ detection. A 2 mM stock solution of copper nitrate was prepared, from which various Cu^2+^ concentrations were obtained by serial dilution. For the fluorescence quenching studies, aqueous solutions of 8PEG-AgNCs and Cu^2+^ with different concentrations were mixed, and further equilibrated for 10 min before the fluorescence spectral measurements. The preliminary investigation indicated that the reaction could reach equilibrium within 10 min. In order to evaluate the selectivity of Cu^2+^ fluorescence detection by using 8PEG-AgNCs nanohybrids, other metal ions such as Hg^2+^, Fe^2+^, Zn^2+^, Mg^2+^, Fe^3+^, Na^+^, Cr^3+^, Mn^2+^, Al^3+^, K^+^ and Pb^2+^ were chosen to be tested and analyzed. All the measurements were conducted at least in triplicate.

### 2.8. Instruments and Measurements

The UV/vis spectra and fluorescence spectra were obtained with a TU-1901 UV–visible spectrometer (Purkinje, Beijing, China) and F-7000 fluorescence spectrophotometer (AXimA-CFR, Hitachi, Japan), respectively. A drop of 8PEG-AgNCs solution was put on a grid and dried for 30 min under an infrared lamp and then the samples were measured on a JEOL JEM-100 CX II TEM (JEOL, Tokyo, Japan) under an accelerating voltage of 100 kV. The high-resolution TEM (HRTEM) test was performed on a HRTEM JEM-3030F under 200 kV. Fluorescence quantum yield of the 8PEG-AgNCs nanohybrid was measured by a Quantaurus-QY absolute photoluminescence quantum yield spectrometer (Hamamatsu, Japan). A dynamic laser scattering (DLS) instrument (BI-90Plus) was used to analyze the size distribution of the 8PEG-AgNCs nanospheres. X-ray photoelectron spectroscopy (XPS) was conducted on an ESCALAB 250 X-ray photoelectron spectrometer (Thermofisher, Waltham, MA, USA) with a monochromatized Al Kα X-ray source (1486.71 eV). Gel Permeation Chromatography (GPC) was performed at 25 °C utilizing a Korean YL9100/YL9130 System with a refractive index detector.

## 3. Results and Discussion

### 3.1. Analysis of the Gelling Process of PEGOMA and PEGOA Hydrogel Templates

**Figure 2:**
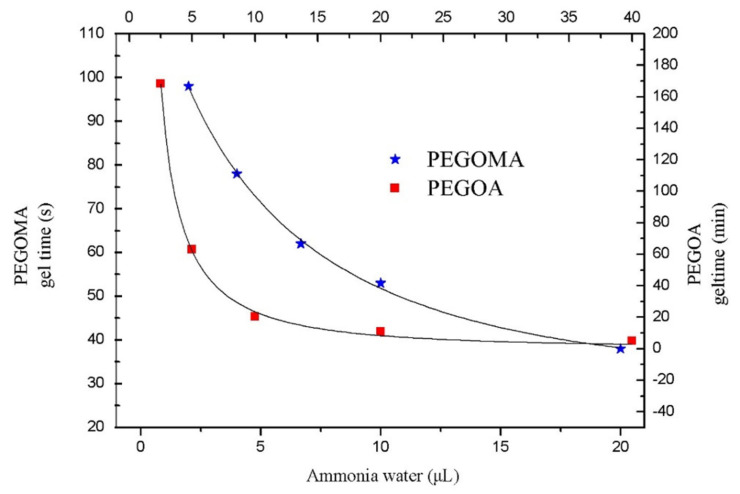
Gelation time changes of PEGOMA and PEGOA hydrogels with the added amount of ammonia water.

This section begins with the properties of the modified 8PEG hydrogels cross-linked with ammonia. The basic gelation properties and gelation principles of PEG hydrogels were initiated to determine the function of eight-armed PEG hydrogels in the process of template synthesis of AgNCs nanomaterials. The study of the gelation properties of hydrogels began with the critical gelation concentration of a hydrogel, which was the critical gelation concentration of a monomer as a sufficient cross-linker. That is to say, it is the lowest solution concentration that can form a gel without fragmentation under the circumstances. Firstly, 150 mg of purified PEGOA and PEGOMA were dissolved in 0.1, 0.2, and 0.1 mL, respectively. An amount of 50 μL of primary water at various concentrations of 0.4, 0.8, 1.6, and 3 mL, etc. was added to ammonia. The critical gel concentration of PEGOA was measured at about 100 mg/mL half an hour after the sonication. In this system, the critical gel concentration of PEGOMA was about 180 mg/mL. After the homogeneous solution, the gels were smooth, transparent and had some brittleness. Take PEGOA dissolved in 0.2 mL water as an example, the gel formed was as follows (Figure 2).

The gelation time of a hydrogel is one of the most important indicators for a hydrogel and can also be used to illustrate the cross-linking process of a hydrogel. PEGOMA hydrogels and PEGOA hydrogels are the most important crosslinking agents. The variation of gelation time of the hydrogel with the addition of cross-linker ammonia is shown in Figure 2. The gelation time of PEGOMA hydrogel is much shorter than that of PEGOA at the same ammonia concentration. The concentration of fixed end-modified un-crosslinked PEG remained unchanged, gradually decreasing from 40 to 2 μL as ammonia was added. The gel time of PEGOMA hydrogel was gradually extended from 38 to 98 s, and the gel time of PEGOA hydrogel from 5 min gradually extended to 168.3 min.

**Figure 3:**
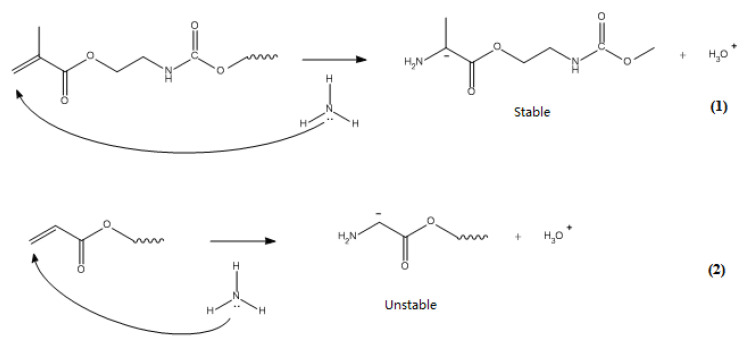
Mechanism of the Michael addition reaction of PEGOMA and PEGOA with ammonia water.

Therefore, the gel time of PEGOMA was much shorter than that of PEGOA. In methacrylate PEG, the methyl group can stabilize the negative charge formed by the Michael addition reaction. This reaction can generate tertiary carbon negative ions before recapturing hydrogen atoms. While the carbon anion can also be stabilized by conjugation, the activation energy of the reaction is low and irreversible. The double bond at the acrylate end is not stabilized by this effect, and the secondary carbon anion generated by the reaction cannot be stabilized, therefore, it cannot be stabilized. The process exists in the formation of PEGOA and can only generate an alkanol-like structure similar to the 1,4 addition. The reaction is slower and has a higher activation energy, and the possible mechanism of the reaction is shown in Figure 3.

**Figure 4:**
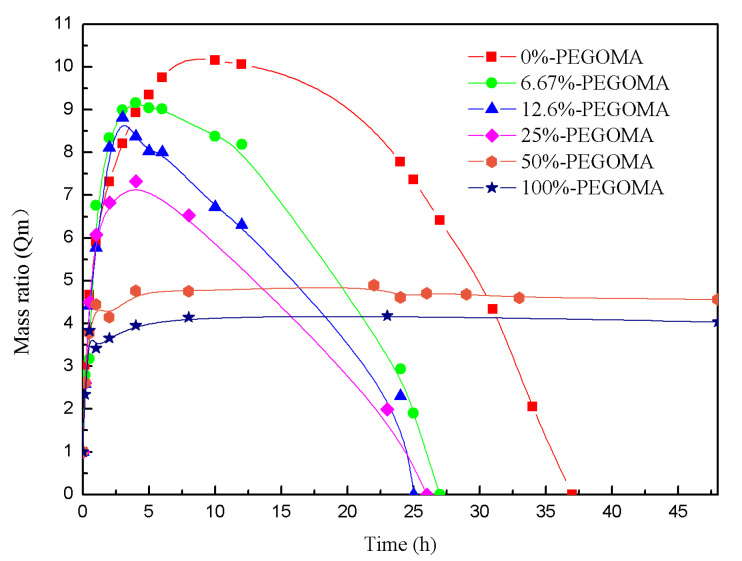
Mass ratio changes of different kinds of polyethylene glycol (PEG) hydrogel template (gels with different PEGOA/PEGOMA blending ratios) with time.

As can be seen from Figure 4, although both are PEG hydrogels, the pure PEGOA hydrogel has a higher water absorption and swelling rate. This indicates that the cross-linking density is greater and the cross-linking yields a more imperfect hydrogel network chain. With the addition of PEGOMA, the mass ratio of hydrogels shows a decreasing trend, indicating that ammonia speeds up the reaction and perfects the formation of the net chain. Another phenomenon observed from Figure 4 is that, when the PEGOMA content is less than 25%, the degradation forms an aqueous solution of nanosilver clusters with fluorescent properties. In addition, when the PEGOMA content is 25–50%, it forms a long-stable hydrogel with fluorescent properties under UV light, and a colorless transparent gel at room temperature. When the PEGOMA content was greater than 50%, there was no fluorescence after the mixture was crosslinked and placed in water for a certain period of time, and only colorless transparent gels were present. In combination with the analysis of the pore size of the gel templates, this may be due to the synergistic effect from the silver ion reduction by the terminal hydroxyl group of the PEG degradation products and the limiting effect of the pore volume within the gel template.

By characterizing the basic hydrogel properties, this section determines that the gel-forming concentration of the hydrogel template used to be 750 mg/mL. It is reasonable for the cross-linking reaction to proceed smoothly, while not having to wait for a long time. The addition of 30 mL of ammonia was sufficient for the complete crosslinking reaction and a short crosslinking time.

### 3.2. Fabrication of 8PEG-AgNCs Nanohybrids

Very recently, a new class of eight-armed star-shaped PEG-based hydrogels (8PEG-NH_3_) has been developed in our lab [40], in which ammonium solution (NH_3_) serves as the crosslinker. They were formed by an amine Michael-type addition reaction between unsaturated carbon–carbon double bonds (acrylate end groups) and reactive amine species. By variation of the crosslinking parameters, the degree of residual functional groups, the swelling degree and the mechanical properties of the resulting gels can be elegantly tuned [41,42]. 

Since the NH_3_ solution functions between two and three in practice, the fully modified eight-armed PEG has a functionality of eight. Compared to the synthesis of PEG hydrogels using a linear PEG (functionality of two) and a cross-linker (functionality of typically two), ammonia-crosslinked eight-armed PEG hydrogels can achieve a denser crosslink density and result in a denser inner pore size of the hydrogel. Theoretically, they could be applied as hyperbranched polymers and as templates for synthetic nanoclusters. Therefore, the strong fluorescent 8PEG-AgNCs hydrogels were formed when mixing NH_3_ solution with an 8PEG precursor containing Ag^+^ ions.

The mechanism of using 8PEG-NH_3_ hydrogel as template to obtain AgNCs could be explained by the synergistic effect of hydroxyl groups from 8PEG as a reduction agent and the nanocages surrounded by crosslinking polymer chains as a nano reactor (cage effect). Ag^+^ ions can be reduced to Ag^0^ clusters by the oxidation of hydroxyl groups from PEG in the absence of any other reducing reagents [43]. The reducing reactivity of PEG can be remarkably increased as the length of the PEG chain increases [44]. Moreover, amidogen can be introduced to the PEG chains through a Michael addition reaction [45], which could chelate with Ag^+^ ions to stabilize AgNCs. Meanwhile, the oxygens on the PEG chains provide coordinate bonds with AgNCs and hence enhance their stability in aqueous solution. The stability was similar to the DNA-templated fluorescent AgNCs, developed by Sharma et al. [25]. 

Nanocages inside the 8PEG gel matrix is another key factor to obtain fluorescent AgNCs. The crosslinking structure of the 8PEG gel matrix has been supposed to result in a “cage effect” (Figure 1), which improves the stability of AgNCs and prevents continued growth of nanoclusters to larger nanoparticles [46]. Mixing the linear PEG (LPEG) solution containing Ag^+^ ion with NH_3_ solution leads to larger plasmonic Ag nanoparticles (LPEG-AgNPs) rather than nanoclusters, this is because linear PEG reacted with NH_3_ cannot form a gel, indicating that “cage effect” plays a crucial role in the formation of AgNCs. Finally, due to the hydrolysis of ester groups in the gel matrix, 8PEG-AgNCs nanohybrids can be released via the gel’s degradation. 

**Figure 5:**
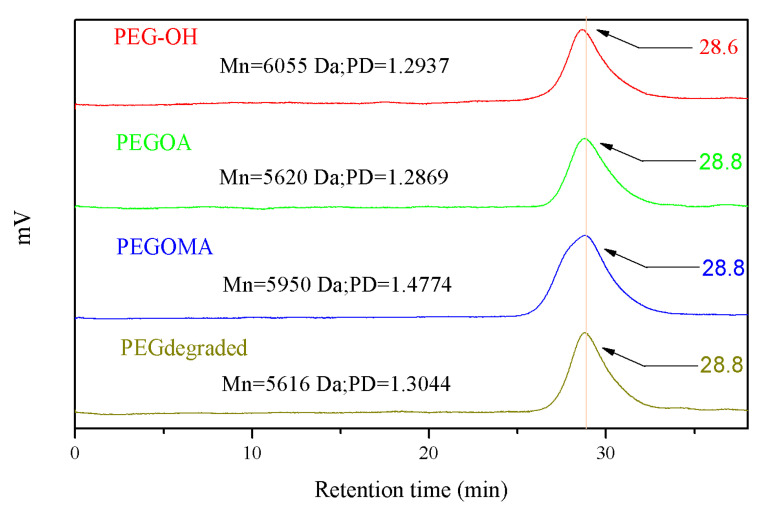
Gel Permeation Chromatography (GPC) analysis of 8PEG-OH, PEGOA, PEGOMA and degradation product of PEGOA-PEGOMA hybrid gel template.

GPC has been utilized to determine the molecular weight of degradation products, which are nearly identical to that of 8PEG polymers. As can be seen from Figure 5, the molecular weight (Mn) of the degradation products of the PEG blended hydrogel was similar to that of the initial polymer. This probably due to the existence of some adsorption interaction between the end-modified groups and the gel column, which increased the retention time. No large change in retention time was observed during the synthesis and degradation process, which indicates that the preparation and degradation of the gel template did not show any molecular weight change or main chain breakage. The larger polydispersity coefficient (PD) of PEGOMA was 1.4774, while the other three were similar, probably due to a small number of ester exchange reactions in PEGOMA. These results suggest the possibility of the total degradation of the gel matrix, as well as the complete releasing of 8PEG-AgNCs, as illustrated in Figure 1.

**Figure 6:**
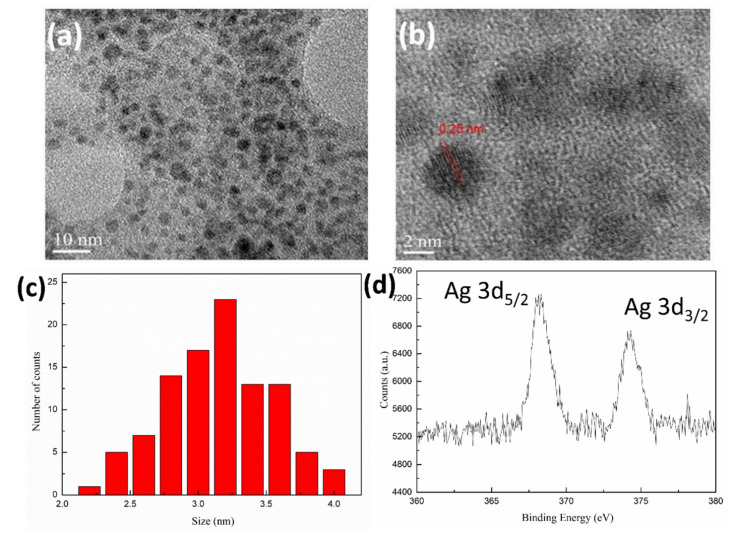
(**a**) High resolution (HR)TEM images of as-synthesized 8PEG-AgNCs nanohybrids, (**b**) Lattice fringes of AgNCs, (**c**) Particle size distribution and (**d**) X-ray photoelectron spectroscopy (XPS) characterization of 8PEG-AgNCs.

HRTEM images of the 8PEG-AgNCs are given in Figure 6a–c. The as-synthesized AgNCs are nearly spherical and well dispersed. The lattice fringes of AgNCs are clearly determined as 2.5 Å corresponding to d(111) (0.18 nm), proving the existence of Ag nano crystals [47]. The average diameter of AgNCs is 3.27 nm (Figure 6c), which is appreciably larger than most of the reported AgNCs. We suggest that is the result of AgNCs being densely covered by PEG chains, accounting for the excellent water solubility and sustainable fluorescence at room temperature.

The XPS wide spectrum of the 8PEG-AgNCs (Figure 6d) shows peaks at 282.2 and 529.5 eV, which are attributed to C 1 s and O 1s from PEG chains, respectively. In addition, two peaks at 368.2 and 374.2 eV are attributed to the binding energy of Ag 3d, in which a 6 eV gap is in agreement with the zero valence of AgNCs [48]. DLS measurements of 8PEG-AgNCs in deionized water confirm a relatively narrow size distribution around 10 nm larger than the TEM diameter, revealing the core-shell structure of 8PEG-AgNCs.

### 3.3. Fluorescent Properties of 8PEG-AgNCs Nanohybrids

**Figure 7:**
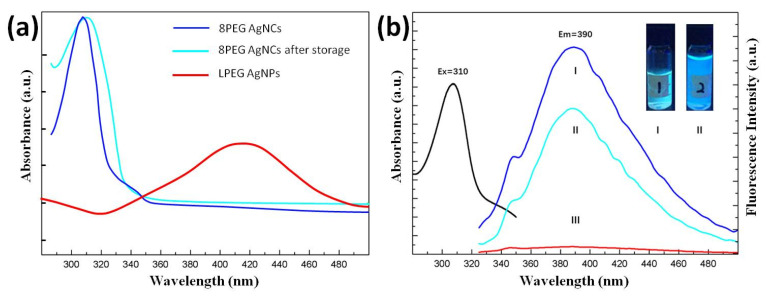
(**a**) UV/vis absorption spectra and (**b**) fluorescence spectra of fresh (Ι), reserved 8PEG-AgNCs solution (ΙΙ) and linear PEG-AgNCs solution (ΙΙΙ). Inset: photographs of fresh (Ι) and reserved 8PEG-AgNCs solution (ΙΙ) under and UV light excitation.

Compared with other templates for the synthesis of AgNCs, the 8PEG material as a template for the preparation of AgNCs has different fluorescence properties in addition to the stable long-term stability of the synthesized AgNCs. The UV/vis absorption spectra and fluorescence spectra of fresh 8PEG-AgNCs, reserved 8PEG-AgNCs solution (stored in the dark, at room temperature for one year) and PEG-AgNPs are shown in Figure 7a,b, respectively. Both fresh and reserved 8PEG-AgNCs present a narrow absorption peak at 310 nm (Figure 7a), without the characteristic plasmon peak of larger AgNCs in the range of 400–500 nm, indicating the formation of AgNCs. On the contrary, the plasmonic peak of AgNCs appear in the spectra of the linear PEG solution. Due to no gel formation in the linear case of PEG, nanoclusters can continually grow to generate large-sized nanoparticles.

After determining the UV absorption peak of the AgNCs, the maximum absorption peak of the fluorescence emission spectrum of the aqueous solution of the AgNCs which passes through the fluorescence spectroscopic characterization can also be determined. Figure 7b shows the spectral characterization of the fluorescence of aqueous solutions of AgNCs synthesized by the PEGOA template method, and the fluorescence of the AgNC solutions. The peak position of the emission spectrum is taken as the maximum fluorescence intensity at this wavelength, which corresponds to its UV absorption spectrum, both at 310 nm.

When excited by UV radiation at 310 nm, both AgNCs solutions present a wide fluorescence emission peak at 393 nm (Figure 7b); for comparison, barely no emission peaks have been found in linear PEG solution, indicating that the nanocages in the gel matrix are essential for the synthesis of the fluorescent AgNCs. The fluorescence quantum yield of 8PEG-AgNCs at λ_ex_ = 310 nm was measured to be 7.6% via using quinine sulfate as the reference. In the inset of Figure 7b, the photographs of fresh and reserved 8PEG-AgNCs in aqueous solution, which emits intense blue fluorescence under a UV lamp, are shown. Moreover, the fluorescence intensity of 8PEG-AgNCs after one year storage only shows 30% attenuation (Figure 7b), suggesting that the AgNCs are well protected by surrounding PEG chains and exhibit high stability.

The fluorescence stability improved from up to three months to more than one year compared to AgNCs. This shows that the degraded 8PEG molecular chains are fully protected against the AgNCs. AgNCs synthesized using 8PEG as a template did not exhibit significant fluorescence emission at any excitation wavelength under the same conditions. This further suggests that the AgNCs synthesized by 8PEG are large nanoparticles. However, the greater cross-linking density leads to smaller gel pores, allowing the synthesis of nanoparticles with fluorescent properties. Moreover, the Stokes shift of the AgNCs was calculated to be 83 nm. Compared to the usual Stokes shifts of hundreds, this value is considerably lower. The fluorescence excitation peak is 310 nm, indicating that this AgNCs has an unusual fluorescence resonance energy transfer, which may have potential applications in fluorescence detection.

### 3.4. Fluorescent Sensor for Detection of Cu^2+^ Ions

**Figure 8:**
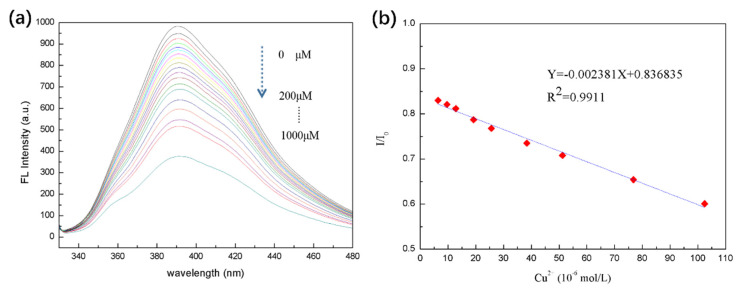
(**a**) Fluorescent response of 8PEG-AgNCs in the presence of various metal ions (λ_ex_ = 310 nm, λ_em_ =393 nm, C_metal_ =100 μM, C_Cu_^2+^ = 100 μM). (**b**) Fluorescence emission spectra of 8PEG-AgNCs upon addition of various quantities of Cu^2+^ (0–1000 μM).

Considering the health issue, in drinking water the safe limit of Cu^2+^ ions has been set at 1.3 ppm (ca. 20 μM) [49]. A low-cost, facile and rapid tracking of Cu^2+^ ions can be achieved via a fluorescent sensor through fluorescence quenching when Cu^2+^ ions contact with 8PEG-AgNCs. The selectivity of the 8PEG-AgNCs (1 mg/L) probe toward Cu^2+^ ions (100 μM), relative to other metal ions (100 μM) has been tested. When adding Cu^2+^ ions into the 8PEG-AgNCs solution, fluorescent intensity decreases by 50%, in contrary to no tremendous fluorescence quenching observed in the presence of other metal ions. Figure 8a reveals that our probes responded toward Cu^2+^ ions at least 20-fold more than a variety of competing metal ions, indicating excellent selectivity of Cu^2+^ detection. 

It can be demonstrated experimentally that the limit of detection of Cu^2+^ ions by the 8PEG-AgNCs prepared by this template method is 50 nM. This is a value of three standard deviations of the burst fluorescence intensity greater than the instrumental detection after the addition of a certain amount. The 8PEG-AgNCs amount was relatively low, indicating that the 8PEG-AgNCs were sensitive to the presence of Cu^2+^. The AgNCs prepared by this template method still had strong fluorescence production at Cu^2+^ ion concentrations higher than 1000 μM. It also sidelines the stability of the prepared 8PEG-AgNCs, with PEG molecular chains wrapped around the surface of the AgNCs to maintain their certain fluorescence properties. 

The fluorescence emission spectra of 8PEG-AgNCs nanohybrids in the presence of Cu^2+^ with varying concentrations are presented in Figure 8b. It was found that the fluorescence intensity decreased gradually, when increasing the concentration of Cu^2+^. Compared with other previous works on Cu^2+^ detection [50], the dense PEG chain protection not only brings about the unusual stability of AgNCs in aqueous solution, but also prevents AgNCs from complete fluorescence quenching; even the concentrations of Cu^2+^ ions reach up to 1000 μM. 

The relative fluorescence intensity I/I_0_ versus the Cu^2+^ concentration up to 100 μM is plotted in Figure 8b, where I_0_ and I are the fluorescence intensities at 393 nm in the absence and presence of Cu^2+^ ions. In the linear range, AgNCs can be used as a fluorescence detection method for Cu^2+^ ions in the range of 5–100 μM, relatively. It is a significant improvement over the conventional Cu^2+^ detection range and is more suitable for practical applications. The detection concentration requirement for Cu^2+^ detection in drinking water is 20 μM. The mechanism of fluorescence bursting of AgNCs for the detection of Cu^2+^ can be based on the following Lineweaver–Burk equation.

$$\frac{F_{0}}{F}\;=\;1\;+\;K_{SV}C_{q}$$

where *F*_0_ and *F* are the fluorescence intensities of the AgNCs in the presence and absence of Cu^2+^, respectively, *K_sv_* is the dynamic bursts constant, and *C_q_* is the concentration of the bursting agent. From the formula, *F*_0_/*F*, if proportional to the concentration of the bursting agent, means that the mechanism of fluorescence bursting is dynamic bursting. Therefore, in this work, the fluorescence burst of the AgNCs may be due to a dynamic burst produced by collisions with Cu^2+^ that transfer energy to the Cu^2+^.

Linear correlation coefficient R^2^ = 0.99 exists between the value of I/I_0_ and the concentration of Cu^2+^ ions over the range 5–100 μM as presented in Figure 7b, satisfying the sensitivity requirement of Cu^2+^ detection for drinking water (20 μmol/L). The limit of detectable response is 50 nM (10 ppb), based on a signal-to-noise ratio of three. The standard copper detection limit in drinking water is 1.3 ppm in US and 2 ppm in Europe and according to the WHO [51]. The limit of detection by 8PEG-AgNCs is much lower than the existing standard Cu^2+^ ion detection limit. These results indicate that the as-prepared 8PEG-AgNCs nanohybrids display excellent selectivity and sensitivity toward Cu^2+^ ions. 

**Figure 9:**
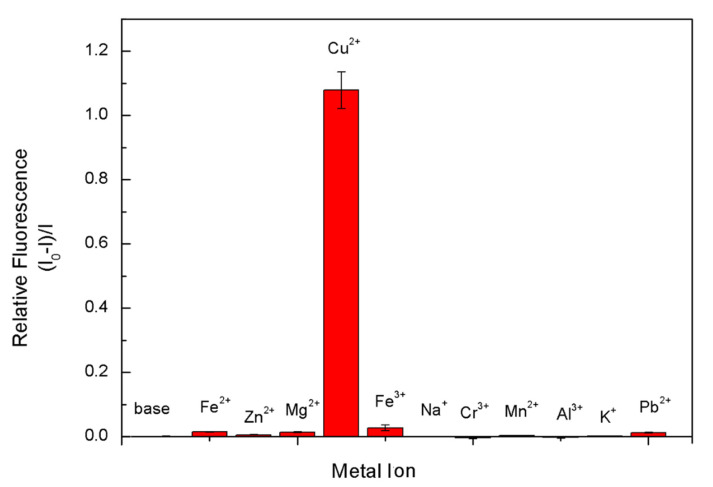
The linear plot of the relative fluorescence intensity (I/I_0_) of 8PEG-AgNCs record at 393 nm versus the concentration of Cu^2+^.

In addition to the detection limit and detection range of fluorescence detection, the selectivity of fluorescence detection is also very important. By dropping the same concentrations of different kinds of metal ions commonly found in water into a solution of AgNCs, it was found that AgNCs were only effective in copper (Figure 9). The fluorescence intensity decreases by more than 50% in the presence of ions, while the addition of other metal ions shows a stronger burst of fluorescence. Only the iron ions showed a small of fluorescence bursting effect, which can be neglected in the actual detection. The Ca^2+^ ions, which were normally not influenced by the fluorescent sensors [47,52], were neglected in this selectivity test. This showed that the AgNCs prepared by the template method have a very high specificity selection for Cu^2+^ ions in the fluorescence detection, and in the real water.

Comparing with other systems for Cu^2+^ ion selectivity, the 8PEG-AgNCs system in our work still has superior features. Kaewnok et al. fabricated a fluorescent sensor based on helicene dye bearing hydrazine. The sensor selectively detected Cu^2+^ ions. The detection limit was 2.6 ppb [53]. Udhayakumari et al. found that 2,3-diaminophenazine, 1,2-diamino-anthraquinone, and 2,4-dinitrophenylhydrazine could work as fluorescent probes for Cu^2+^ ion selective detection. The phenomena were due to the paramagnetic effect and inhibition of photoinduced electron transfer [54]. In another work, Jung et al. developed a coumarin-based fluorogenic probe as a fluorescent chemosensor. This material exhibited high selectivity for Cu^2+^ ions due to the fluorescent quenching mechanism [55]. The sensor system developed in this work is biodegradable and echo-friendly. In the work of Lee et al., they developed a colorimetric sensor based on a DNAzyme/AuNP for detecting uranium(VI). When exposed to uranium(VI), the solution color changed from purple to red. This was attributed to the fact that uranium(VI) led to the de-aggregation of the functionalized AuNPs. The detection limit was 12 ppb [56]. The limit of detection in this work is lower than this system. A series of comparison was provided in Table 1.

In some of the Ag-based systems for ion detection, the mechanism behind them most of the time lays in color change. Firdaus et al. developed a AgNP-based colorimetric sensor for the detection of mercury(II). By adding mercury(II), the solution color changed from yellow to brown. The detection limit was 0.85 μM (170 ppb). This sensor has high selectivity for mercury(II) among interfering ions [59]. Some of the work focused on Raman signal change. Liang et al. found that AgNPs along with antigen Cr-EDTA-bis(trimethylsilyl)acetamide (BSA) in immunoassays could be utilized to detect chromium(III) in water. The Raman signal of chromium(III) was decreased when a mixture of chromium(III) solution and AgNPs was flown through the immunoassay. The detection limit was 0.01 ppt to 0.01 ppb [60]. 

However, the fluorescent sensor provided a lower detection limit and much higher sensitivity. Liu et al. synthesized hydrophilic zinc-doped AgInS2 (AIZS) quantum dots (QDs) through a mini-emulsion/solvent evaporation technique. The AIZS QDs were successfully applied for the selective detection of Cu^2+^ ions. The detection range was from 0 to 340 μM and the limit of detection (LOD) was 27.3 nM [52]. Shang et al. reported poly(methacrylic acid) (PMAA)-templated AgNCs could be utilized to detect Cu^2+^ ions. This was due to the quenching effect of functionalized AgNCs and Cu^2+^ ions. The sensing range was from 1.0 × 10^−8^ M to 6.0 × 10^−6^ M and the detection limit was 8 nM [47]. According to the similarity of our AgNCs and those in the literature, we assumed that the mechanism behind was fluorescent quenching.

When the system was complexed with copper atoms, the sensitivity was even better. Su et al. developed a sensor system based on 3-mercaptopropionic acid (MPA) and DNA-Cu/Ag nanoclusters (NCs) for Cu^2+^ ion detection. This was attributed to the fact that the Cu^2+^ ions resulted in the oxidation of MPA to form a disulfide compound. This compound led to recovery of the fluorescence of the DNA-Cu/AgNCs. The detection range was from 5 to 200 nM. The limit of detection was 2.7 nM [58]. This complex combination will be considered in our next working system for enhancing the detection sensitivity. 

After the preparation of AgNCs by this facile template method in this work, the relationship between the degree of fluorescence deactivation and the amount of copper ions added was studied. The results illustrated the capability of 8PEG-AgNCs as fluorescent sensor for Cu^2+^ ions. By comparison with other literature, the mechanism of the fluorescence burst between the template synthesized AgNCs and Cu^2+^ ions is assumed to be a fluorescent quenching mechanism. The fluorescence detection test showed that the synthesized AgNCs are suitable for the online monitoring of Cu^2+^ ions in real environments.

## 4. Conclusions

In summary, we presented a novel, facile and low-cost method for one-pot, in situ synthesis of highly fluorescent AgNCs via a gel-mediated process. Well-dispersed AgNCs coated with PEG presents extreme long-term stability. It has been proved that nanocages inside the gel matrix play a crucial role in the formation of nanoclusters, due to the volume restriction effect. The synthesis of AgNCs using eight-armed PEG templates has the advantages of a simple method, no need for reducing agents, high stability and good water solubility. In the next stage, the as-synthesized AgNCs utilized for Cu^2+^ detection reveal excellent selectivity and sensitivity. Study of the relationship between the degree of fluorescence extinction and the amount of Cu^2+^ ions added indicated the corresponding detection limits, linear range and linear correlation. The linear detection of Cu^2+^ ions by AgNCs synthesized by this template method ranged from 5–100 μmol/L. This novel gel-mediated strategy displays a unique combination of advantages, namely simple and versatile preparation, long-term stability, controllable nanocluster growth, and the degradability of the template. These advantages will be highly interesting in the nanotechnology field.

## Figures and Tables

**Table 1:** Some examples of sensors for Cu^2+^ ion selectivity.

Materials	Cu^2+^ Selectivity	Detection Limit	Proposed Mechanism	Ref.
Helicene dye bearing hydrazine	good	2.6 ppb	photoinduced electron transfer	[53]
2,3-diaminophenazine, 1,2-diamino-anthraquinone, 2,4-dinitrophenylhydrazine	good	0.15 × 10^−9^ M/L	paramagnetic effect	[54]
Coumarin-based fluorogenic probe bearing the 2-picolyl unit	good	0.5 µM	quenching mechanism	[55]
Zinc-doped AgInS2 quantum dots	good	27.3 nM	electron transfer	[52]
Poly (methacrylic acid) (PMAA)-templated AgNCs	good	8 nM	quenching mechanism	[47]
DNA–AgNCs	/	1.4 mM	marvelous fluorescent enhancement in the presence of histidine	[57]
DNA-Cu/AgNCs	good	2.7 nM	static quenching mechanism	[58]
8PEG-AgNCs	good	50 nM	quenching mechanism	This work

## References

[1] Chakraborty I., Pradeep T. (2017). Atomically precise clusters of noble metals: Emerging link between atoms and nanoparticles. Chem. Rev..

[2] Baral A., Basu K., Ghosh S., Bhattacharyya K., Roy S., Datta A., Banerjee A. (2017). Size specific emission in peptide capped gold quantum clusters with tunable photoswitching behavior. Nanoscale.

[3] Shang L., Dong S., Nienhaus G.U. (2011). Ultra-small fluorescent metal nanoclusters: Synthesis and biological applications. Nano Today.

[4] Yu X., Liu W., Deng X., Yan S., Su Z. (2018). Gold nanocluster embedded bovine serum albumin nanofibers-graphene hybrid membranes for the efficient detection and separation of mercury ion. Chem. Eng. J..

[5] Yu X., Wang Z., Su Z., Wei G. (2017). Design, fabrication, and biomedical applications of bioinspired peptide-inorganic nanomaterial hybrids. J. Mater. Chem. B.

[6] Zhang W., Lin D., Wang H., Li J., Nienhaus G.U., Su Z., Wei G., Shang L. (2017). Supramolecular self-assembly bioinspired synthesis of luminescent gold nanocluster-embedded peptide nanofibers for temperature sensing and cellular imaging. Bioconjugate Chem..

[7] Ritchie C.M., Johnsen K.R., Kiser J.R., Antoku Y., Dickson R.M., Petty J.T. (2007). Ag nanocluster formation using a cytosine oligonucleotide template. J. Phys. Chem. C.

[8] New S., Lee S., Su X. (2016). DNA-templated silver nanoclusters: Structural correlation and fluorescence modulation. Nanoscale.

[9] Sharma J., Yeh H.-C., Yoo H., Werner J.H., Martinez J.S. (2010). A complementary palette of fluorescent silver nanoclusters. Chem. Commun..

[10] Mei L., Teng Z., Zhu G., Liu Y., Zhang F., Zhang J., Li Y., Guan Y., Luo Y., Chen X. (2017). Silver nanocluster-embedded zein films as antimicrobial coating materials for food packaging. ACS Appl. Mater. Interfaces.

[11] Ma Y., Shen X.-F., Liu F., Pang Y.-H. (2020). Colorimetric detection toward halide ions by a silver nanocluster hydrogel. Talanta.

[12] Ershov B., Janata E., Henglein A., Fojtik A. (1993). Silver atoms and clusters in aqueous solution: Absorption spectra and the particle growth in the absence of stabilizing Ag^+^ ions. J. Phys. Chem..

[13] Henglein A. (1989). Small-particle research: Physicochemical properties of extremely small colloidal metal and semiconductor particles. Chem. Rev..

[14] Udayabhaskararao T., Pradeep T. (2013). New protocols for the synthesis of stable Ag and Au nanocluster molecules. J. Phys. Chem. Lett..

[15] Kumar S., Bolan M.D., Bigioni T.P. (2010). Glutathione-stabilized magic-number silver cluster compounds. J. Am. Chem. Soc..

[16] Kitazawa H., Albrecht K., Yamamoto K. (2012). Synthesis of a dendrimer reactor for clusters with a magic number. Chem. Lett..

[17] Xu H., Suslick K.S. (2010). Sonochemical synthesis of highly fluorescent Ag nanoclusters. ACS Nano.

[18] Adhikari B., Banerjee A. (2010). Short-peptide-based hydrogel: A template for the in situ synthesis of fluorescent silver nanoclusters by using sunlight. Chem. A Eur. J..

[19] Yu J., Patel S.A., Dickson R.M. (2007). In vitro and intracellular production of peptide-encapsulated fluorescent silver nanoclusters. Angew. Chem. Int. Ed..

[20] Copp S.M., Bogdanov P., Debord M., Singh A., Gwinn E. (2014). Base motif recognition and design of DNA templates for fluorescent silver clusters by machine learning. Adv. Mater..

[21] Tan R., He Y., Zhu Y., Xu B., Cao L. (2003). Hydrothermal preparation of mesoporous TiO_2_ powder from Ti(SO_4_)_2_ with poly (ethylene glycol) as template. J. Mater. Sci..

[22] Shen Z., Duan H., Frey H. (2007). Water-soluble fluorescent Ag nanoclusters obtained from multiarm star poly (acrylic acid) as “molecular hydrogel” templates. Adv. Mater..

[23] Dai Y., Hu X., Wang C., Chen D., Jiang X., Zhu C., Yu B., Qiu J. (2007). Fluorescent Ag nanoclusters in glass induced by an infrared femtosecond laser. Chem. Phys. Lett..

[24] Huang Z., Pu F., Lin Y., Ren J., Qu X. (2011). Modulating DNA-templated silver nanoclusters for fluorescence turn-on detection of thiol compounds. Chem. Commun..

[25] Sharma J., Rocha R.C., Phipps M.L., Yeh H.-C., Balatsky K.A., Vu D.M., Shreve A.P., Werner J.H., Martinez J.S. (2012). A DNA-templated fluorescent silver nanocluster with enhanced stability. Nanoscale.

[26] Shang L., Dong S. (2008). Facile preparation of water-soluble fluorescent silver nanoclusters using a polyelectrolyte template. Chem. Commun..

[27] Barthel M.J., Angeloni I., Petrelli A., Avellini T., Scarpellini A., Bertoni G., Armirotti A., Moreels I., Pellegrino T. (2015). Synthesis of highly fluorescent copper clusters using living polymer chains as combined reducing agents and ligands. ACS Nano.

[28] Shang G., Li C., Wen G., Zhang X., Liang A., Jiang Z. (2016). A new silver nanochain SERS analytical platform to detect trace hexametaphosphate with a rhodamine S molecular probe. Luminescence.

[29] Schmidt E.W. (2001). Hydrazine and Its Derivatives: Preparation, Properties, Applications, 2 Volume Set.

[30] Choi S., Dickson R.M., Yu J. (2012). Developing luminescent silver nanodots for biological applications. Chem. Soc. Rev..

[31] Wang S., Meng X., Das A., Li T., Song Y., Cao T., Zhu X., Zhu M., Jin R. (2014). A 200-fold quantum yield boost in the photoluminescence of silver-doped AgxAu_25_-x nanoclusters: The 13th silver atom matters. Angew. Chem..

[32] Zinchenko A., Miwa Y., Lopatina L.I., Sergeyev V.G., Murata S. (2014). DNA hydrogel as a template for synthesis of ultrasmall gold nanoparticles for catalytic applications. ACS Appl. Mater. Interfaces.

[33] Liu W., Zhang X., Wei G., Su Z. (2018). Reduced graphene oxide-based double network polymeric hydrogels for pressure and temperature sensing. Sensors.

[34] Liu W., Zhang W., Yu X., Zhang G., Su Z. (2016). Synthesis and biomedical applications of fluorescent nanogels. Polym. Chem..

[35] Zhang J., Xu S., Kumacheva E. (2005). Photogeneration of fluorescent silver nanoclusters in polymer microgels. Adv. Mater..

[36] Chakraborty I., Udayabhaskararao T., Pradeep T. (2012). Luminescent sub-nanometer clusters for metal ion sensing: A new direction in nanosensors. J. Hazard. Mater..

[37] Ma B., Dang W., Yang Z., Chang J., Wu C. (2020). MoS2 Nanoclusters-based biomaterials for disease-impaired wound therapy. Appl. Mater. Today.

[38] Roy S., Banerjee A. (2011). Amino acid based smart hydrogel: Formation, characterization and fluorescence properties of silver nanoclusters within the hydrogel matrix. Soft Matter.

[39] Guo S.R., Gong J.Y., Jiang P., Wu M., Lu Y., Yu S.H. (2008). Biocompatible, luminescent silver@ phenol formaldehyde resin core/shell nanospheres: Large-scale synthesis and application for in vivo bioimaging. Adv. Funct. Mater..

[40] Zhang Z., Li Q., Yesildag C., Bartsch C., Zhang X., Liu W., Loebus A., Su Z., Lensen M.C. (2018). Influence of network structure on the crystallization behavior in chemically crosslinked hydrogels. Polymers.

[41] Zhang Z., Loebus A., de Vicente G., Ren F., Arafeh M., Ouyang Z., Lensen M.C. (2014). Synthesis of poly (ethylene glycol)-based hydrogels via amine-michael type addition with tunable stiffness and postgelation chemical functionality. Chem. Mater..

[42] Ren F., Yesildag C., Zhang Z., Lensen M.C. (2017). Functional PEG-hydrogels convey gold nanoparticles from silicon and aid cell adhesion onto the nanocomposites. Chem. Mater..

[43] Liz-Marzán L.M., Lado-Touriño I. (1996). Reduction and stabilization of silver nanoparticles in ethanol by nonionic surfactants. Langmuir.

[44] Luo C., Zhang Y., Zeng X., Zeng Y., Wang Y. (2005). The role of poly (ethylene glycol) in the formation of silver nanoparticles. J. Colloid Interface Sci..

[45] Kumar R., Chaudhary P., Nimesh S., Chandra R. (2006). Polyethylene glycol as a non-ionic liquid solvent for Michael addition reaction of amines to conjugated alkenes. Green Chem..

[46] Zheng J., Dickson R.M. (2002). Individual water-soluble dendrimer-encapsulated silver nanodot fluorescence. J. Am. Chem. Soc..

[47] Shang L., Dong S. (2008). Silver nanocluster-based fluorescent sensors for sensitive detection of Cu(II). J. Mater. Chem..

[48] Wang C., Huang Y. (2014). Facile preparation of fluorescent Ag-clusters-chitosan-hybrid nanocomposites for bio-applications. New J. Chem..

[49] Liu S., Tian J., Wang L., Zhang Y., Qin X., Luo Y., Asiri A.M., Al-Youbi A.O., Sun X. (2012). Hydrothermal treatment of grass: A low-cost, green route to nitrogen-doped, carbon-rich, photoluminescent polymer nanodots as an effective fluorescent sensing platform for label-free detection of Cu(II) ions. Adv. Mater..

[50] Karaoglu K., Yilmaz F., Menteşe E. (2017). A new fluorescent “turn-off” coumarin-based chemosensor: Synthesis, structure and Cu-selective Fluorescent sensing in water samples. J. Fluoresc..

[51] Dalmieda J., Kruse P. (2019). Metal cation detection in drinking water. Sensors.

[52] Liu Y., Zhu T., Deng M., Tang X., Han S., Liu A., Bai Y., Qu D., Huang X., Qiu F. (2018). Selective and sensitive detection of copper (II) based on fluorescent zinc-doped AgInS2 quantum dots. J. Lumin..

[53] Kaewnok N., Petdum A., Sirirak J., Charoenpanich A., Panchan W., Sahasithiwat S., Sooksimuang T., Wanichacheva N. (2018). Novel Cu^2+^-specific “Turn-ON” fluorescent probe based on [5] helicene with very large Stokes shift and its potential application in living cells. New J. Chem..

[54] Udhayakumari D., Velmathi S., Sung Y.-M., Wu S.-P. (2014). Highly fluorescent probe for copper (II) ion based on commercially available compounds and live cell imaging. Sens. Actuators B Chem..

[55] Jung H.S., Kwon P.S., Lee J.W., Kim J.I., Hong C.S., Kim J.W., Yan S., Lee J.Y., Lee J.H., Joo T. (2009). Coumarin-derived Cu^2+^-selective fluorescence sensor: Synthesis, mechanisms, and applications in living cells. J. Am. Chem. Soc..

[56] Lee J.H., Wang Z., Liu J., Lu Y. (2008). Highly sensitive and selective colorimetric sensors for uranyl (UO_2_^2+^): Development and comparison of labeled and label-free DNAzyme-gold nanoparticle systems. J. Am. Chem. Soc..

[57] Zhou Y., Zhou T., Zhang M., Shi G. (2014). A DNA–scaffolded silver nanocluster/Cu_2_+ ensemble as a turn-on fluorescent probe for histidine. Analyst.

[58] Su Y.-T., Lan G.-Y., Chen W.-Y., Chang H.-T. (2010). Detection of copper ions through recovery of the fluorescence of DNA-templated copper/silver nanoclusters in the presence of mercaptopropionic acid. Anal. Chem..

[59] Firdaus M.L., Fitriani I., Wyantuti S., Hartati Y.W., Khaydarov R., McAlister J.A., Obata H., Gamo T. (2017). Colorimetric detection of mercury (II) ion in aqueous solution using silver nanoparticles. Anal. Sci..

[60] Liang J., Liu H., Lan C., Fu Q., Huang C., Luo Z., Jiang T., Tang Y. (2014). Silver nanoparticle enhanced Raman scattering-based lateral flow immunoassays for ultra-sensitive detection of the heavy metal chromium. Nanotechnology.

